# Acceptability of Mobile App–Based Motivational Interviewing and Preferences for App Features to Support Self-Management in Patients With Type 2 Diabetes: Qualitative Study

**DOI:** 10.2196/48310

**Published:** 2024-03-06

**Authors:** Sungwon Yoon, Haoming Tang, Chao Min Tan, Jie Kie Phang, Yu Heng Kwan, Lian Leng Low

**Affiliations:** 1 Health Services and Systems Research Duke-NUS Medical School Singapore Singapore; 2 Centre for Population Health Research and Implementation SingHealth Regional Health System Singapore Singapore; 3 Duke-NUS Medical School Singapore Singapore; 4 Internal Medicine Residency SingHealth Residency Singapore Singapore; 5 Post-Acute and Continuing Care Outram Community Hospital Singapore Singapore; 6 SingHealth Duke-NUS Family Medicine Academic Clinical Program Singapore Singapore

**Keywords:** mobile health, motivational interviewing, diabetes, self-management, health coaching, acceptability, application, management, type 2 diabetes, communication, patient barrier, healthy behavior, feedback, visualization, hybrid model

## Abstract

**Background:**

Patients with type 2 diabetes mellitus (T2DM) experience multiple barriers to improving self-management. Evidence suggests that motivational interviewing (MI), a patient-centered communication method, can address patient barriers and promote healthy behavior. Despite the value of MI, existing MI studies predominantly used face-to-face or phone-based interventions. With the growing adoption of smartphones, automated MI techniques powered by artificial intelligence on mobile devices may offer effective motivational support to patients with T2DM.

**Objective:**

This study aimed to explore the perspectives of patients with T2DM on the acceptability of app-based MI in routine health care and collect their feedback on specific MI module features to inform our future intervention.

**Methods:**

We conducted semistructured interviews with patients with T2DM, recruited from public primary care clinics. All interviews were audio recorded and transcribed verbatim. Thematic analysis was conducted using NVivo.

**Results:**

In total, 33 patients with T2DM participated in the study. Participants saw MI as a mental reminder to increase motivation and a complementary care model conducive to self-reflection and behavior change. Yet, there was a sense of reluctance, mainly stemming from potential compromise of autonomy in self-care by the introduction of MI. Some participants felt confident in their ability to manage conditions independently, while others reported already making changes and preferred self-management at their own pace. Compared with in-person MI, app-based MI was viewed as offering a more relaxed atmosphere for open sharing without being judged by health care providers. However, participants questioned the lack of human touch, which could potentially undermine a patient-provider therapeutic relationship. To sustain motivation, participants suggested more features of an ongoing supportive nature such as the visualization of milestones, gamified challenges and incremental rewards according to achievements, tailored multimedia resources based on goals, and conversational tools that are interactive and empathic.

**Conclusions:**

Our findings suggest the need for a hybrid model of intervention involving both app-based automated MI and human coaching. Patient feedback on specific app features will be incorporated into the module development and tested in a randomized controlled trial.

## Introduction

Type 2 diabetes mellitus (T2DM) is a leading cause of mortality and disability. Globally, 537 million adults have diabetes, and it is projected to increase to 783 million by 2045 [1]. In Singapore, 1 in 3 adults are at risk of developing diabetes in their lifetime [2]. The prevalence of T2DM will increase from 14.2% in 2022 to 25% in 2050, highlighting the urgent need for developing effective management strategies for patients with T2DM [3].

Self-management has been found to be effective in enhancing clinical and behavioral outcomes of patients with T2DM [4]. However, research indicates that self-management in patients with T2DM is inadequate due to the lack of adherence to healthy behavior and medications [5]. This is concerning because poorly controlled T2DM results in increased incidence of life-threatening complications such as neuropathy, retinopathy, amputation, and cardiovascular disease [6-8]. Patients’ knowledge deficit, lack of motivation toward behavior change, and inadequate self-discipline have been identified as main patient-related barriers to effective self-management [9-11].

Motivational interviewing (MI) is a patient-centered and goal-oriented communication method that can address patient barriers and promote positive health behavior changes [12]. Central to MI is assisting a patient to resolve inner state of ambivalence by expressing empathy, avoiding argumentation, developing discrepancy, and supporting self-efficacy [13,14]. Evidence suggests that MI holds promise for improving self-management of T2DM [15]. Several systematic reviews and meta-analysis of randomized controlled trials (RCTs) have found that MI-based interventions contributed to not only a reduction in hemoglobin A_1c_ value but also improvements in self-management skills, dietary behaviors, and emotional well-being, albeit some of these positive results were not sustained long term [12,16,17].

Although existing literature provides important insight, the vast majority of studies used face-to-face or telephone-based MI interventions [18,19]. With the growing adoption and penetration of smartphones, automated MI techniques powered by artificial intelligence (AI) on mobile devices may offer effective motivational support to patients, complementing the traditional model of in-person counseling. In addition, the delivery of MI using AI could allow more sustainable scaling up and implementation of MI in clinical practice [20]. However, there is little evidence supporting the use of mobile app–based MI in improving health outcomes of patients with T2DM. Furthermore, no study explored the acceptability of app-based MI among patients with T2DM as end users [21]. Incorporating end-user feedback into the design of MI would be essential to improving the effectiveness of the MI intervention for patients with T2DM.

We have developed a mobile app EMPOWER that performs remote monitoring and education of patients with T2DM through AI-powered personalized nudges. The clinical effectiveness of the EMPOWER app is being tested through an ongoing RCT [22]. The addition of an MI module into the EMPOWER app has been planned for improved T2DM management as a follow-on intervention. This study aimed to explore the perspectives of patients with T2DM on the acceptability of app-based MI in routine health care and collect patient feedback on MI module features to inform future interventions.

## Methods

### Study Design

The study adopted a qualitative research method involving semistructured interviews.

### Participant Recruitment

Eligibility criteria included patients who had a diagnosis of T2DM, aged 40 years and older, and had no cognitive impairment that prohibits normal conversation. Patients with gestational diabetes or serious diabetes-related complications were excluded. Eligible patients were recruited from polyclinics, which provide subsidized comprehensive and integrated public primary care services in Singapore. Patients were purposively recruited in terms of age (40-49, 50-59, and 60-69 years old) and educational attainment (university and above, diploma, secondary school, and primary and below) to ensure a diversity of opinions from July 2022 to November 2022. Previous studies have demonstrated that age and education levels influence app use [23-26].

### Data Collection

A semistructured interview guide was developed based on the review of relevant literature and pilot-tested with 3 participants (data included). Topics included current diabetes management, confidence and importance of behavior changes, acceptability of MI in general and app-based MI in combination or the absence of health coaches, preferences for the mode of MI delivery, and usefulness of MI module features. In this study, app-based MI includes delivery of MI through rule-based techniques and machine learning techniques, without the involvement of humans. To assess participants’ confidence and importance of behavior changes, we used the 0-10 ruler (numerical rating scale), which is recommended by Miller and Rollnick [13,14]. These rulers have been validated for tobacco cessation [27]. To facilitate specific feedback from participants, we used a mock-up app wireframe similar to the appearance of a proposed module wireframe built on a transtheoretical model [28] and self-determination theory [29]. The wireframe included features such as rulers of importance and confidence, self-reflection and change talk with goal setting, tracking of progress and nudging, backup plan writings, educational resources, and gamification and rewards, along with a summary page of goals and achievements that may be shared with health care providers. The wireframe focused on 3 areas to promote diabetes self-management: diet, physical activity, and medication adherence. These module features had been iterated over time as the interview progressed. All interviews were conducted via videoconferencing in English and Mandarin by interviewers trained in qualitative research. The interviews lasted approximately 60 minutes in duration. Field notes were taken during the interviews.

### Data Analysis

All interviews were audio recorded and transcribed verbatim. Transcripts were thematically analyzed [30]. Coding categories were developed based on the following steps: familiarizing data by reading transcripts line by line, developing a coding frame to apply to the whole data set, attributing data to individual codes, collating codes into themes, and interpreting them through meaning and connections. Each transcript was coded by 3 coders (HT, CW, JL). Agreement regarding the coding frame and category refinement was achieved via discussions and reflexive reviews of the previous codes and emergence of new themes. The code categories and themes were subsequently reviewed by the study team to ensure that the codes reflect the major themes that emerge from the data. The NVivo 12 software (Lumivero) was used for analysis. Data collection and analysis were conducted in an iterative manner until thematic saturation was accomplished. To ensure transparency, rigor, and trustworthiness, we used a detailed audit trail, member checking, and reflexivity at each step [31]. Participant feedback was not sought due to difficulty in recontacting patients.

### Ethical Considerations

The study was approved by the SingHealth Centralized Institutional Review Board (CIRB 2022/2031). Participants provided verbal informed consent prior to study commencement. The study team maintained data confidentiality by redacting personally identifiable information from interview transcripts and generating unique study identifiers, which were linked to participant identifiable information through a password-protected file. Participants were reimbursed SGD $50 to defray the cost of their participation in this research.

## Results

### Characteristics of Participants

A total of 33 patients participated. Data saturation was achieved with 30 interviews. The mean age of the participants was 56 years. Approximately 70% (23/33) were male and 85% were Chinese. The majority were working full-time (20/33, 61%), and more than half (28/33, 85%) of the participants attained secondary education and above. Participants had comorbid health conditions such as hypertension and hyperlipidemia. Median motivational ruler ratings of importance and confidence were 8.5 and 7, respectively (Table 1).

Findings were presented by 3 major areas: perceptions of MI as part of routine health care, receptivity toward app-based MI, and feedback on app-based MI module features.

**Table 1:** Participant characteristics (N=33).

Participant characteristic		Value
Age (years), mean (range)		56 (42-66)
**Sex, n (%)**		
	Male	23 (70)
	Female	10 (30)
**Ethnicity, n (%)**		
	Chinese	28 (85)
	Non-Chinese (Malay, Indian, others)	5 (15)
**Employment status, n (%)**		
	Full-time	20 (61)
	Part-time	7 (21)
	Retired or unemployed	6 (18)
**Education, n (%)**		
	University and above	9 (27)
	Diploma	11 (33)
	Secondary school	8 (24)
	Primary and below	5 (15)
**Medical condition^a^, n (%)**		
	Type 2 diabetes mellitus	33 (100)
	Hypertension	21 (64)
	Hyperlipidemia	17 (52)
Importance to change (1-10), median (range)		8.5 (5-10)
Confidence to change (1-10), median (range)		7.0 (1.5-10)

^a^Participants may have multiple conditions.

### Perceptions of MI as Part of Routine Health Care

#### MI Serving as a Mental Reminder to Build Confidence and Motivation

By and large, participants were open to the idea of MI. They stated that something would have to be done to improve their current state of self-management. This is because their motivation to maintain healthy behaviors was often attenuated by a host of challenges. Participants believed that MI could offer them the encouragement and mindset required to overcome the “mental barriers,” which are psychological challenges that hinder their consistent engagement in healthy behavior, such as a lack of self-discipline and motivation.

MI would be good to overcome mental barriers. MI can serve as a check-in mechanism to remind me of my progress and how to improve [my behavior]. So even when I am tired, I will still make an effort to exercise.

Participant #31, male

Other participants noted that additional assistance from MI would enable them to learn new knowledge and build confidence to improve self-management skills.

I would like to have somebody that I can talk to because he or she will understand what I could eat or what I could do, that will help lower my cholesterol or improve diabetes.

Participant #19, male

#### MI as a Complementary Care Model to Existing Health Care Services

Participants felt that MI would be a useful tool to address problems they experienced in busy primary care clinics. Many expressed issues of care discontinuity at length. For example, being unable to consistently see the same provider undermined their interest in listening to advice. Frustrations related to receiving conflicting health advice from different providers seemed to further compound trusting relationship and willingness to change health behaviors. Hence, they saw MI as a care model that would complement the existing services.

Let’s just say that most of the time, doctors just throw you a chunk of information and then you're supposed to go home and digest it. Then, digestion or indigestion is another issue…so I am open to it [MI]. It’s something that will benefit me.

Participant #22, female

#### Perceived Behavioral Control Leading to Reluctance to MI

Despite many being interested in trying the MI, some patients expressed a strong desire to self-manage their conditions and change behaviors. Some felt confident in their ability to manage conditions, while others reported already making changes and preferred self-management at their own pace.

Actually, I’m very independent, doing things on my own. I don't really listen to any counsellor. I know the direction that I wanted to head to...So, I got to do it on my own. I prefer to do it on my own.

Participant #03, female

#### Time Constraints and Competing Demands Diminishing Interest in MI

A host of competing demands was mentioned by several participants as something that would diminish their interest in MI-based coaching sessions. MI was characterized as useful, but engaging in MI was considered a physical and cognitive burden over many more important responsibilities related to family and employment that may take priority.

If a counsellor wants to motivate me, if I got the time [to listen] and if it’s what I want, I will do. Though I am very open, my time is really not enough so I don’t think I will participate [in-person].

Participant #08, male

### Receptivity Toward App-Based MI Using AI for Self-Management

#### Perceived Convenience for Access

By and large, participants agreed that mobile app–based MI would be convenient compared with in-person sessions given greater flexibility in terms of access and scheduling. Those who expressed unwillingness to try MI due to competing priorities welcomed the potential of app-based MI as an ideal alternative to face-to-face MI.

Well, for my case, I would prefer an app [based MI] because I can do this like, anywhere. During my lunchtime, I can do it while I am at my work.

Participant #32, male

#### Enabling Person-Centered Advice

Some participants expressed a preference for app-based MI over in-person MI where they often received health advice that was less individualized and potentially difficult to adopt. They felt that the app-based MI’s ability to tailor individual needs and circumstances in an ongoing self-management journey would help foster motivation through timely and pertinent guidance.

Diet wise, I would prefer more app-based MI because it can be individualized. I have been advised not to eat this and that [from healthcare professionals]. I get frustrated because it's like someone keeps telling me to avoid certain food, which then becomes my own problem…I’d like to get advice through app on what I can eat or why I can't eat.

Participant #11, female

#### Appreciation of Anonymity

Participants in favor of app-based MI expressed their feeling of discomfort about in-person consultation for fear of being judged or being told off. They felt that they would be more guarded and less relaxed when they were asked to share their lifestyle behaviors and self-management.

Because sometimes face-to-face you want to say something, but you cannot articulate. That's something I am worried about, like offending someone. So, this [app] is better. If I am not happy with what I will say, I don’t have to mention immediately in the app.

Participant #10, male

#### Concerns About the Lack of Human Touch

Participants at the same time expressed concerns about lack of authentic human contact and insufficient social connections between the app and the users. A few participants highlighted the importance of verbal and nonverbal gestures and cues in social conversation that could play an important role in engaging and motivating patients. They were worried that the app-based MI may not be able to build a relational foundation that in-person session could offer.

I mean the kind of personal touch in MI must be done face-to-face. And even in counselling, I believe sometimes tapping on the shoulder, saying something softly, could change the mood as well.

Participant #18, male

#### Limited Digital Literacy to Adopt App-Based MI

Some older participants who were less receptive to app-based MI raised issues about the navigation of various features. They were worried that the app-based MI would not be easily learned and adopted due to technical complexity.

I’m not so into this because different apps are always giving me problems. I have to find the code and speak to people [to learn how to use it]. It's quite frustrating for some of us older folks who are not IT savvy.

Participant #14, female

### Participant Feedback on App-Based MI Module Features

#### Overall Module Design and Interface

##### Simplicity and Ease of Navigation

Participants suggested that the module interface should be easy to navigate to ensure that users with limited digital experience could follow the instructions. On average, participants were willing to use the MI module for 10 minutes with the flexibility of responding to 3 or more MI-related questions. The suggested interval between using the MI module ranged from once a week to once every 6 months. They would like the motivational prompts to be concise and relevant to positive behaviors based on completed tasks.

I will say that for the design, you might want to make it simple for beginners. You can ask people 10 questions but for others who are not tech-savvy, you can just ask three questions. If someone has a lot of things to tell you, you can ask like 20 questions.

Participant #05, male

##### More Visualization Tools to Foster Motivation

The necessity for additional visuals, beyond graphics, was stressed by many participants. Participants expressed that clear visualization would enable them to closely monitor their progress, make necessary adjustments, and change behavior, which ultimately fosters their motivation.

I would prefer seeing, you know, some charts to indicate where I am, so after a certain period, I will know whether I am on the right track. So, a graph or whatever chart will help me. I like more direct outcomes and I want to see them soon.

Participant #01, male

##### Inclusion of a Human Health Coach as Opposed to Being Solely Automated

It was commonly viewed that competent health coaches should be accessible through the app, although they may not be required frequently. The health coach would support the patient’s ongoing efforts to achieve their goals, especially when dealing with complex matters that cannot be addressed by the app alone. This is particularly crucial during the initial stage of using the app, as users may encounter challenges that require immediate guidance and assistance from health coaches.

I would like the health coach to be available on the app. The app may be more for daily tracking, right? Then if the health coach, face-to-face, maybe once a month, can talk to me about what my progress is, to give more professional advice, I think that will help me.

Participant #31, male

#### Specific Module Features

##### Goal Setting and Change Talk

**Figure 1:**
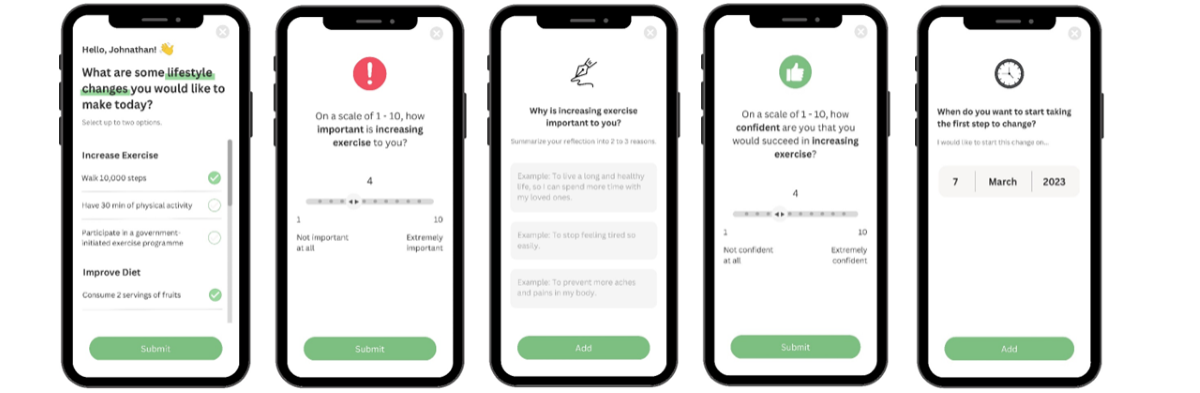
Goal setting and Change Talk. The goal setting feature includes Change Talk, importance and confidence rulers, reasons for change and goal countdown to foster self-reflection on capabilities, intrinsic motivation, and relatedness.

**Figure 2:**
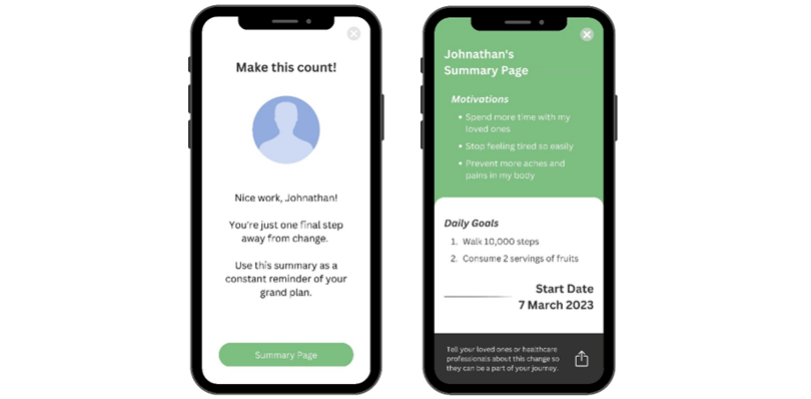
Summary of motivations and goals. The summary page serves to reinforce the patient’s autonomy and intrinsic motivation. It can be shared with a human health coach remotely to improve a sense of relatedness.

The initial wireframe included a goal setting (allowing users to set right-sized and attainable goals), diary (prompting users to reflect on reasons for change), rulers of importance and confidence (user’s level of motivation and self-efficacy), and goal countdown (enabling users to determine a start date) to encourage the patient’s self-reflection and autonomy. While participants appreciated the ability to set personal goals for behavior change, they suggested the goal setting function to be more specific and direct with some examples (eg, take the stairs and take 0% sugar drinks). Importantly, many desired to receive more guidance to ensure the attainment of those goals (Figures 1 and 2).

The goal setting will help me achieve what I want to achieve, by giving me better vision and future target, so once I have achieved that target, I can move on to the next target. As I move on, I achieve certain milestones, then from there, it sort of motivates me to continue.

Participant #16, male

Personally, the best solution for me is, daily when I open the app, it can tell me what I need to do instead of writing so many journals in this app. Better ask me what I want to change and tell me what I can do to improve. I just need a very straightforward instruction.

Participant #31, male

##### Educational Resources

**Figure 3:**
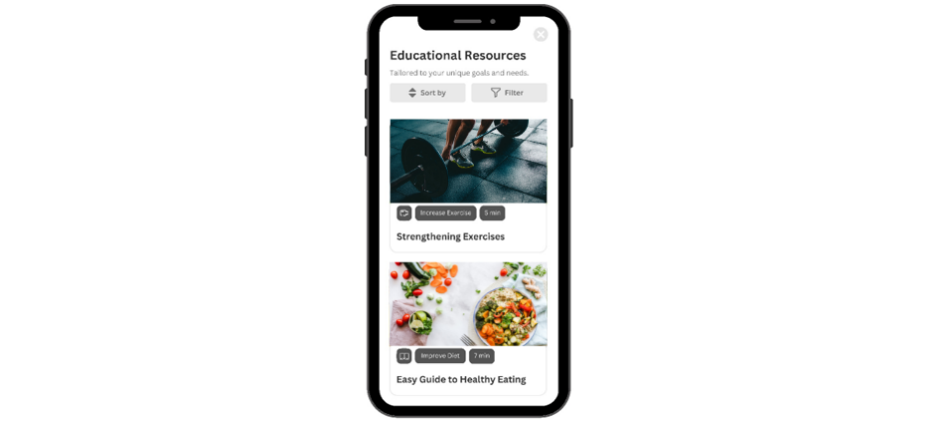
Educational resources. Tailored educational resources based on goals increase patient competence and intrinsic motivation.

Health education materials were designed to improve autonomous motivation by providing tailored educational resources and guidance. Participants wished to have more multimedia resources that they found easier to understand compared with textual information. Participants would like to receive specific health information based on personal goals and needs (eg, definition of refined carbohydrates; Figure 3).

I would like to see more live ones. I don’t like to read a lot of words or look at cartoons. Sometimes, those things are really misleading, and you don’t understand what they are talking about, like some exercises I saw in graphic forms.

Participant #04, female

##### Tracking and Nudges Adaptable to Behavioral Data

**Figure 4:**
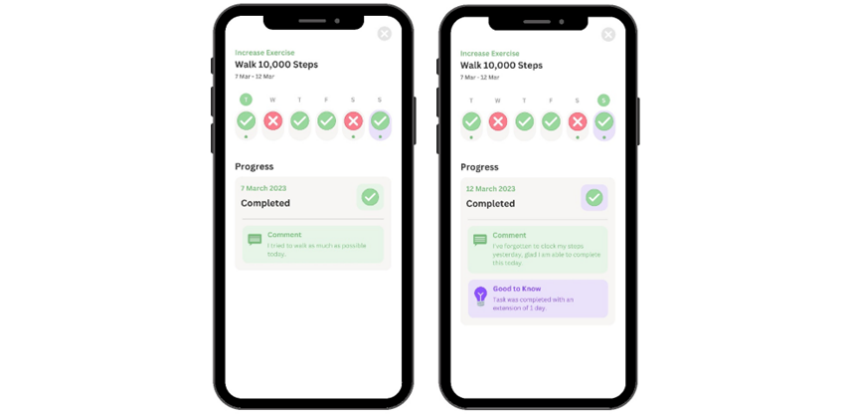
Progress tracking and nudging. Progress tracking and nudging (reminders) with multiple measures of success improve patient competence and self-efficacy for sustained engagement.

The wireframe presented algorithm-based notifications that support patient competence and self-efficacy to continue engaging in health behavior. Participants liked the idea of nudging to help motivate the app users and felt that daily prompts would be an important reminder. In addition to daily prompts, they would like to review weekly and monthly health tasks. Participants desired a 2-way conversational feature where the prompts can be interactive and empathic with different types or tones of encouragement (Figure 4).

Reminders will help pay attention to your diabetes management, because you might forget and go back to old ways of eating sweet things. But if someone tells me that you must cut your sugar intake, then maybe it will remind me that I shouldn’t be taking so much sugar. It’s like having someone to remind you of…a motivating force.

Participant #015, male

I like the motivational prompts to be like a two-way communication. So instead of simply telling me ‘Today, you have zero hours of walking’, the reminder can say ‘have you done this already today? Why was it not done yet? Why are you so busy?’ A gentle reminder. Just like talking to your friend who understands me.

Participant #01, male

##### Gamification and Rewards

**Figure 5:**
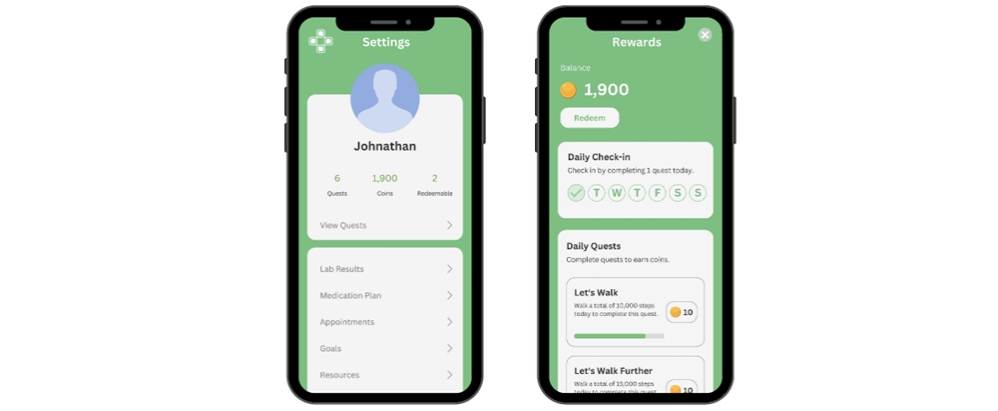
Gamification and rewards. Gamified challenges and rewards enhance patient competence and autonomous motivation through fun activity.

Features of gamified challenges and rewards were included in the wireframe to increase patient competence and intrinsic motivation. Participants suggested incremental incentives for cumulative days engaged or the number of health tasks completed to make sure that individuals could stay motivated (Figure 5).

…Rewarding will encourage people to change behaviors. If you can exchange points for a voucher, that’s a very good idea, and in addition to step counts, if there are other tasks to increase your points, such as healthy eating, that will motivate people.

Participant #25, male

Lastly, participants acknowledged that MI via a mobile app may not be as effective in addressing their personal concerns as receiving MI from human coaches. However, they expected the MI module to offer advice that would be as clear and pertinent as the one provided by health care providers.

I understand the MI through app cannot replace a human, but I’m hoping that it will be better than a chatbot and as human as possible… Just like when you go to a doctor, they give their direct opinions. Certain predefined answers on chatbots at times are not relevant to my concerns.

Participant #30, male

## Discussion

### Principal Findings and Comparison With Prior Work

This study sought to explore the perspectives of patients with T2DM on the acceptability of MI and app-based MI as part of routine health care and their preferences on MI module features. Most technology-delivered adaptations of MI relied on texting or web-based interventions [32]. To the best of our knowledge, 2 studies used mobile apps for MI, focusing on encouraging behavior change [21] and reducing risky alcohol use [33]. Therefore, our study offers unique perspectives on app-based and AI-enabled MI for T2DM self-management.

In our study, participants in general saw MI as a mental reminder to increase motivation and a potentially complementary care model that allows more opportunities to reflect on and alter their management of T2DM. Despite general openness to MI as part of routine health care, our findings indicate that there was a desire to manage their own condition and behaviors by some participants without having life choices being interfered with by the introduction of MI. This sense of reluctance to MI could stem from the lack of understanding of the principles and core strategies of MI given that none of the participants experienced MI. Literature shows that patients with T2DM preferred to have the autonomy to make decisions about their own management of condition based on personal values, and to avoid external pressures that may influence their decision-making process [34,35]. Recent studies on AI-powered chatbot for brief MI also revealed that there were common perceptions of MI chatbots as less intrusive and less threatening to autonomy compared with their human counterparts [36,37]. Therefore, when implementing an MI intervention in routine clinical care, more efforts should be made on patient education to ensure that patients are adequately informed of the concept, main techniques and benefits of MI, and the difference between MI and a traditional consult model. In addition, the interaction model of MI should provide patients with a sense of independence and autonomy, create ample opportunities to express themselves, and establish reciprocal feedback to empower patients to exercise their self-determination [38].

While the idea of incorporating technology into the delivery of MI was novel, participants were generally receptive to the app-based MI given that app-based MI can be accessed remotely. Notably, app-based MI was seen by many as providing a more relaxed atmosphere for open sharing without having the fear of being judged by their health care providers. This finding echoes prior research that individuals receiving technology-enabled MI appreciated nonjudgmental interaction with a simulated counselor, underscoring the significance of patient-centered reflections and guiding for a change [18,39]. However, participants also expressed reservations regarding the lack of human touch with the app, which could potentially undermine the therapeutic relationship between the provider and the patient. Systematic reviews indicate that MI interventions using technology tended to pay less attention to relational and interpersonal components of MI despite technology-delivered MI’s marked advantages to face-to-face counseling [19,39]. In addition to the limited relational contact, technology can bring its own set of challenges to some patients due to the lack of digital literacy as shown in our study. To foster relational emphasis of MI, our app development will adopt a hybrid model that will consist of automated MI delivered through an app supplemented by human health coaching (which can be through an app, texting, or telephone call). A summary page of goals and achievements can be tracked by a human health coach for further discussion with patients who require additional MI support in a time-efficient manner. Improving digital literacy of patients would be imperative to increasing eventual uptake of technology-enabled MI.

In line with existing literature [21,40,41], participants valued tailored goal setting features that support individual autonomy and choice. At the same time, there were concerns about the ability to reach the goal and longer-term engagement. To sustain motivation via a mobile app, participants requested for features of flexible and ongoing supportive nature such as the visualization of milestones, use of multimedia tailored to their specific needs, and communication tools that are interactive and empathic. Indeed, studies suggest that technology-powered MI interventions involving imagery, carefully designed chatbots and embodied conversational agents as a companion in decision-making and branching algorithms customized to individual motivations could be potentially effective in changing target behaviors [36,42,43]. These efforts will be considered in the current or future version of our MI intervention to improve user experience and patient outcomes. Another important input from participants was the provision of incremental rewards based on goals and gamified challenges for motivation-enhancing activity. Although gamification features are found to increase user engagement and experience of competence [21,44], evidence is sparse regarding its impact on cognitive engagement in behavioral changes. Future research is warranted to assess the effectiveness of digital gamification vis-à-vis nongame mechanism on behavior change in MI interventions for patients with T2DM.

### Strengths and Limitations

The strength of this study lies in its emphasis on cocreation of app-based MI and its optimal implementation with purposively sampled patients with T2DM, which provided a diversity and richness of end users’ perspectives.

This study has a few limitations. Participants were recruited from public primary care clinics, and hence their responses may not represent the range of health care services used by patients with T2DM. With the high median rating of importance to change (8.5) and high median rating of confidence to change (7.0) among the participants in this study, it is possible that the voluntary nature of participation might have introduced a selection bias, with patients who were motivated to change behaviors being more prone to participate. Although we sought to recruit a balanced sample, there was limited representation of female and Indian or Malay participants in our multiethnic population. In addition, previous studies have shown that MI may increase the self-efficacy of participants [45-47]. However, we did not assess the self-efficacy of participants in this qualitative interview as there is no conclusive evidence regarding the sustained effect of MI delivery on self-efficacy. Lastly, because we used a mock-up wireframe of MI features, participant feedback may have been limited to the features presented during the interviews.

### Conclusions

This study examined the acceptability of app-based MI and user preferences on MI module features through qualitative interviews with patients with T2DM to inform the development of module content and optimal implementation of app-based interventions. Our findings revealed general openness to app-based MI. Yet, concerns were raised regarding potential compromise of patient autonomy in self-care and lack of meaningful human engagement. To address these concerns, more consideration should be given to patient education on the core principles and benefits of MI and a hybrid model of intervention involving both automated MI and human health coaching. Specific participant feedback will be incorporated into the app and tested through a pragmatic RCT.

## Abbreviations

AI: artificial intelligence
MI: motivational interviewing
RCT: randomized controlled trial
T2DM: type 2 diabetes mellitus
